# Population genetic analysis of the recently rediscovered Hula painted frog (*Latonia nigriventer*) reveals high genetic diversity and low inbreeding

**DOI:** 10.1038/s41598-018-23587-w

**Published:** 2018-04-03

**Authors:** R. G. Bina Perl, Eli Geffen, Yoram Malka, Adi Barocas, Sharon Renan, Miguel Vences, Sarig Gafny

**Affiliations:** 1 Division of Evolutionary Biology, Zoological Institute, Braunschweig University of Technology, Mendelssohnstr. 4, 38106 Braunschweig, Germany; 2 School of Marine Sciences, Ruppin Academic Center, Michmoret 40297, Israel; 3 School of Zoology, George S. Wise Faculty of Life Sciences, Tel Aviv University, Tel Aviv 69978, Israel; 4 Israel Nature and Parks Authority, 3 Am Ve’Olamo Street, Jerusalem 95463, Israel; 5 San Diego Zoo’s Institute for Conservation Research, 15600 San Pasqual Valley Road, Escondido, CA 92027 USA; 6 Wildlife Conservation Research Unit, Department of Zoology, University of Oxford, Recanati-Kaplan Centre, Tubney House, Abingdon Road, Tubney, Oxfordshire OX13 5QL UK

## Abstract

After its recent rediscovery, the Hula painted frog (*Latonia nigriventer*) has remained one of the world’s rarest and least understood amphibian species. Together with its apparently low dispersal capability and highly disturbed niche, the low abundance of this living fossil calls for urgent conservation measures. We used 18 newly developed microsatellite loci and four different models to calculate the effective population size (N_e_) of a total of 125 Hula painted frog individuals sampled at a single location. We compare the N_e_ estimates to the estimates of potentially reproducing adults in this population (N_ad_) determined through a capture-recapture study on 118 adult Hula painted frogs captured at the same site. Surprisingly, our data suggests that, despite N_ad_ estimates of only ~234–244 and N_e_ estimates of ~16.6–35.8, the species appears to maintain a very high genetic diversity (H_O_ = 0.771) and low inbreeding coefficient (F_IS_ = −0.018). This puzzling outcome could perhaps be explained by the hypotheses of either genetic rescue from one or more unknown Hula painted frog populations nearby or by recent admixture of genetically divergent subpopulations. Independent of which scenario is correct, the original locations of these populations still remain to be determined.

## Introduction

The Hula painted frog (*Latonia nigriventer*), endemic to a small (~6.5 km²), extensively modified region in northern Israel, gained notoriety by being the first amphibian species worldwide to be declared extinct^1^. Its disappearance was linked to the draining of the Lake Hula marshes in the 1950s, which also caused the disappearance of 118 other animal species from this area^2^. After its unexpected rediscovery in 2011^3^, Perl *et al*.^4^ confirmed that the Hula painted frog is a localised species with presumably low dispersal abilities and elusive habits, occurring in apparently low densities and bound to permanent and comparatively deep water bodies. Overall, juvenile and tadpole proportions were low, even though females of this species may lay several hundreds of eggs^4^. These observations suggest that present habitat conditions may not be optimal for the remnant population, possibly leading to a temporary recruitment failure. These findings also highlight the importance of immediate conservation actions for the Hula painted frog, which is still listed as Critically Endangered by the International Union for Conservation of Nature^5^.

Habitat fragmentation, modification and destruction have long been recognised as adversely affecting species composition and diversity, and leading to an increased rate of species extinction^6–12^. In addition, habitat alterations and concomitant population subdivision may increase the effect of random genetic drift and, thus, accelerate the loss of genetic variation in populations^13–15^. A small population size may promote inbreeding and, thereby, lead to reduced viability and fecundity (inbreeding depression) of individuals^16–18^. Both mechanisms can considerably diminish the population’s future adaptability to environmental changes^19^. While the preservation of remaining habitat is probably the key aspect for ensuring the survival of any species in the wild^20–22^, conservation efforts targeting small, remnant populations should also be geared towards maintaining the genetic diversity, and thus, the adaptive potential within these populations^23,24^.

Effective population size (N_e_) and effective number of breeders (N_b_) are two important parameters that play crucial roles in predicting the extinction risks of populations due to endogenous stochastic effects (demographic and genetic stochasticity) or exogenous stochastic effects (natural catastrophes and environmental stochasticity)^25,26^. Besides, other population genetic parameters (e.g. allele frequencies and richness, heterozygosity or inbreeding coefficient) have become increasingly important in conservation planning for threatened species and have been widely used to assess and monitor their genetic variability^27–31^.

The high level of polymorphism of microsatellites has made them particularly useful markers for genetic studies focusing on population estimates and structures, even for closely related individuals^32^. The feasibility of microsatellite genotyping from buccal swab sampling in amphibians^33^, less invasive than many other sampling methods, adds to their attractiveness for conservation studies. Capture-recapture data, on the other hand, have been extensively used to infer census sizes and population movements since the late 19^th^ century. The conjunction of both genetic^34,35^ and demographic based methods has the advantage that the benefits of each approach are combined and the drawbacks of each compensated^36–38^.

In the present study, we combine microsatellite and capture-recapture data on the Hula painted frog in order to estimate (i) the effective population size (N_e_), (ii) the number of potentially reproducing adults (N_ad_) as well as (iii) the relationships among individuals. Therefore, individuals were captured at the single known sizeable population, which was only recently discovered (December 2013). This population is situated outside the protected boundaries of the Hula Nature Reserve, and currently has little protection from habitat destruction and other detrimental anthropogenic effects. Although a few specimens were discovered inside the Hula Nature Reserve, up to the present we were not able to locate a sizeable and stable population there.

## Results

### Allelic diversity

All 18 newly developed microsatellite loci amplified successfully (amplification success: 99.9–100%) and the PCR products were polymorphic (Table 1) and consistently scorable. Allele numbers ranged from five to 12 and mean Shannon’s diversity index was high (2.397). We therefore concluded that these loci were suitable for estimating the size and genetic diversity of the Hula painted frog population (Table 1). We neither found large allele dropouts or null alleles (estimated null allele frequencies were < 0.05), nor did we detect scoring errors in our microsatellite data set. Our test for linkage disequilibrium with subsequent Bonferroni correction (*P* ≤ 0.00033) revealed, however, that 120 out of 153 pairs of loci (78%) were linked. In addition, we found that one out of 18 loci (6%; loci LAT8) was significantly deviant from Hardy Weinberg equilibrium after Bonferroni correction (*P* ≤ 0.0028). Due to the evidence of linkage between most loci, we further investigated this issue in the nine individuals captured within the Hula Nature Reserve. No linkage disequilibrium was detected for any pairwise combination of loci in these individuals (*P* values ranged from 0.0029 to 0.997).

**Table 1:** Genetic parameter estimates for 18 microsatellite loci for the Hula painted frog population under study. Loci significantly deviant from Hardy Weinberg equilibrium after Bonferroni correction (*P* ≤ 0.0028) are marked with asterisk (*). Abbreviation key: N, sample size; A, number of alleles per locus; ^s^H, Shannon’s diversity index; H_O_, observed heterozygosity; H_E,_ expected heterozygosity; F_IS_, inbreeding coefficient.

Locus	N	A	^s^H	H_O_	H_E_	F_IS_
LAT1	125	6.000	2.050	0.792	0.734	−0.079
LAT2	122	10.000	2.915	0.803	0.853	0.058
LAT3	125	7.000	1.570	0.568	0.531	−0.070
LAT4	125	7.000	2.624	0.888	0.822	−0.081
LAT5	125	6.000	1.770	0.640	0.665	0.037
LAT6	123	10.000	3.111	0.911	0.877	−0.039
LAT7	125	7.000	2.367	0.808	0.788	−0.026
LAT8*	125	12.000	3.200	0.896	0.874	−0.026
LAT9	125	9.000	2.351	0.784	0.758	−0.035
LAT10	122	6.000	2.168	0.811	0.736	−0.103
LAT11	123	5.000	1.702	0.602	0.639	0.058
LAT12	120	9.000	2.468	0.733	0.743	0.013
LAT13	125	9.000	2.573	0.736	0.790	0.069
LAT14	125	6.000	2.004	0.728	0.670	−0.040
LAT17	125	9.000	2.675	0.832	0.802	−0.038
LAT18	119	11.000	2.485	0.697	0.711	0.019
LAT19	119	6.000	2.244	0.748	0.758	0.014
LAT20	124	8.000	2.872	0.903	0.856	−0.056
Mean	125	7.944	2.397	0.771	0.756	−0.018

### Population genetic structure

**Figure 1:**
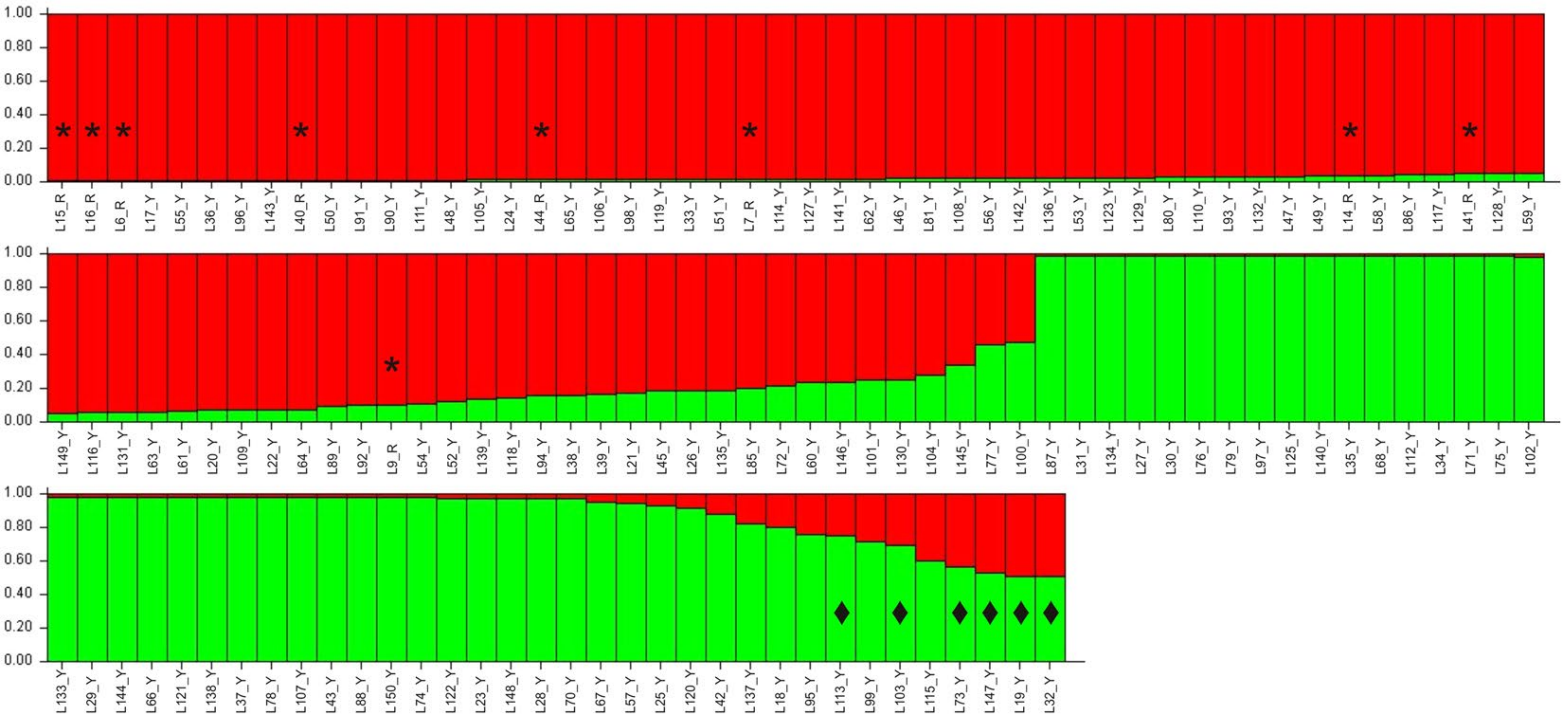
Estimated genetic clustering (*K* = 2) of 134 Hula painted frog individuals captured at two close-by locations in northern Israel as obtained using Bayesian analysis implemented in STRUCTURE. Each bar represents an individual; * = individuals captured within the Hula Nature Reserve (individuals captured outside the reserve are unmarked); ♦ = individuals assigned to the other cluster using the Likelihood Relatedness analysis as implemented in ML-RELATE.

**Figure 2:**
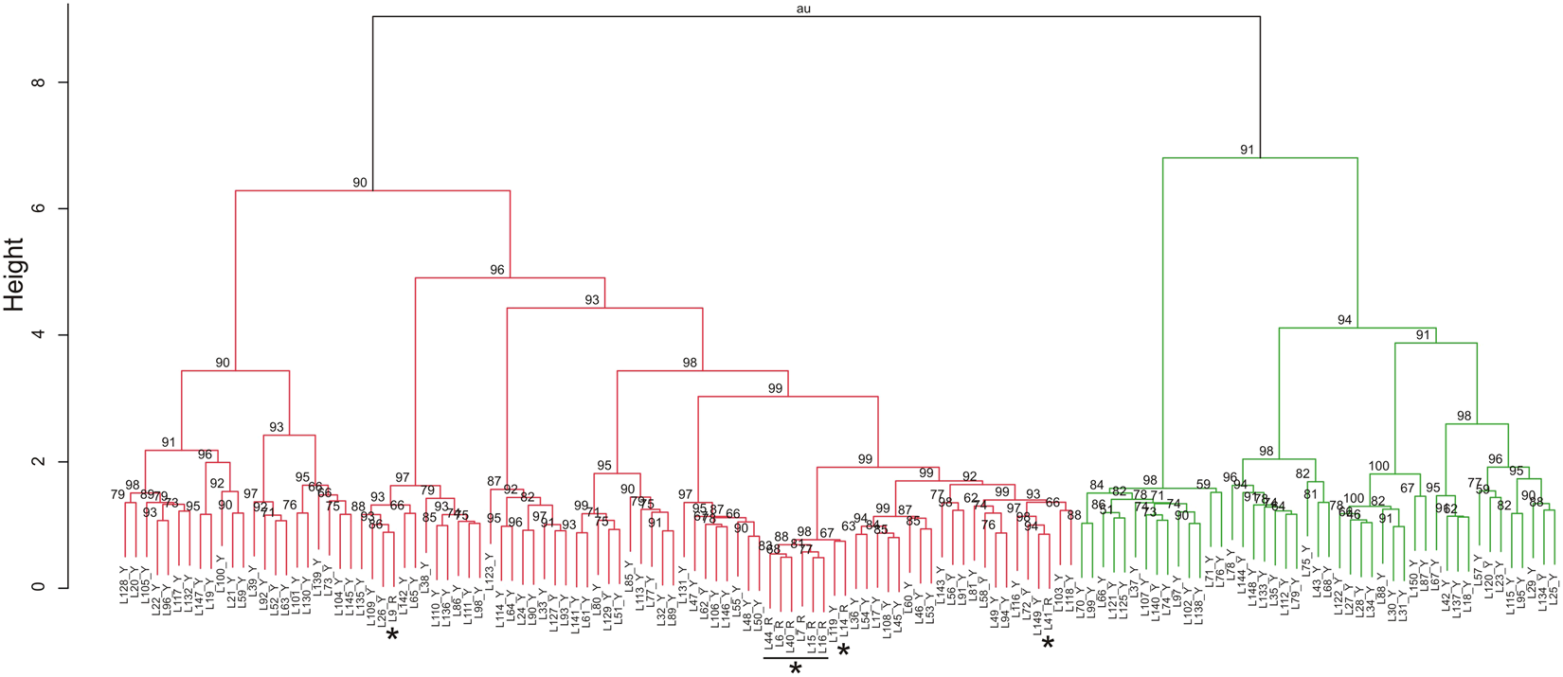
Dendrogram showing the relationship among 134 Hula painted frog individuals captured at two close-by locations in northern Israel. Hierarchical clustering was performed on the likelihood relatedness output obtained by ML-RELATE. Approximately unbiased (au) *P* values (%), as computed by the R package pvclust, are given for each node in the dendrogram (distance: euclidean, cluster method: ward.D2). Colours of branches were chosen to match the clusters obtained by STRUCTURE analysis (see Fig. 1); * = individuals captured within the Hula Nature Reserve (individuals captured outside the reserve are unmarked).

Our STRUCTURE analysis revealed an optimal cluster solution of *K* = 2 clusters (Table 2, Fig. 1) that was to 99.9% supported by the estimated LR among individuals as calculated by ML-RELATE (Fig. 2). Only for six out of 134 individuals both programs produced slightly different results (Fig. 1). However, both programs consistently assigned the nine individuals captured within the Hula Nature Reserve to the same sub-cluster (Figs 1 and 2). A subsequent calculation of the genetic differences confirmed a significant partitioning of genetic variation between the two clusters (F_ST_ = 0.074; *P* > 0.001).

**Table 2:** Inference of the population structure of the Hula painted frog based on the Bayesian analysis of 134 individuals. Abbreviation key: *K*, number of assumed populations; Reps, number of MCMC iterations; Δ*K*, ad hoc statistic based on the change in the log probability data between successive *K* values^72^.

*K*	Reps	Mean LnP(*K*)	Stdev LnP(*K*)	Ln′(*K*)	|Ln″(*K*)|	Δ*K*
1	10	−8593.44	0.3273	—	—	—
2	10	−8115.74	0.6433	477.7	239.51	372.3403
3	10	−7877.55	14.7528	238.19	96.31	6.528254
4	10	−7735.67	38.9997	141.88	31.5	0.807699
5	10	−7625.29	44.2559	110.38	—	—

Among all captured individuals, COLONY identified 143 pairs of individuals as HS dyads and 133 pairs as FS/PO dyads. Our ML-RELATE calculations confirmed 88% of the HS and 84% of the FS/PO assignments (Table 3). Of the nine individuals captured within the Hula Nature Reserve, COLONY only identified two individuals as having relationships to individuals of the location outside the reserve. While one individual from the reserve was identified as a half-sibling to two individuals outside the reserve, the other individual was identified as having a parent-offspring relationship to one individual and being a half-sibling to ten individuals outside the reserve. However, we found eight of the nine individuals captured within the reserve to also be closely related to each other (HS or FS assignments). These COLONY results were fully supported by the ML-RELATE analysis. The results of an additional run in COLONY, where all assigned family clusters included captured juveniles, suggested at least 20 different successful mating pairs during 2012 through 2014.

**Table 3:** Comparison of kinship reconstruction analyses for the Hula painted frog performed by COLONY and ML-RELATE. Abbreviation key: HS, half-sibling; FS, full-sibling; PO, parent offspring.

# of possible pairs	Relationship		Program
	HS	FS/PO	
8911	143	133	COLONY
	126	112	ML-RELATE
	88%	84%	Overlap

### Population size

Given the total number of sampled adults (N = 118), all N_e_ estimates for the investigated Hula painted frog population were relatively low (16.6–35.8; Table 4), independent of the method used. In three out of four cases, the 95% confidence intervals (CI) were comparatively narrow (Table 4), suggesting relatively precise estimates. Despite the extremely low N_e_ values, we found the Hula painted frog population to exhibit a comparatively high genetic diversity. Overall, expected and observed heterozygosities of loci ranged from 0.568 to 0.911 and 0.531 to 0.877, respectively (Table 1). In addition, the number of alleles at each locus (ranged 5–12) and the allelic diversity (ranged 1.570–3.200) were high, whereas the inbreeding coefficient for each locus was very low (ranged −0.103–0.069; Table 1), and significantly lower than expected by chance (*P* value for F_IS_ within samples = 0.003).

**Table 4:** Effective population size estimates (N_e_) for the Hula painted frog population under study as calculated by four different methods meeting different assumptions. CI, confidence interval.

Method	N_e_	CI		Software
		Lower	Upper	
Sibship assignment	31.0	20	53	COLONY
Linkage disequilibrium	21.8	19.8	24.1	LDNE
Molecular coancestry	16.6	7.4	29.4	NeEstimator
Heterozygote excess	35.8	15.7	∞	NeEstimator

Both capture-recapture estimates of N_ad_ were substantially higher than the N_e_ estimates (236 individuals with CAPWIRE, and 244 individuals with MARK; Table 5) and, with exception of the values obtained from the heterozygote excess method, the CIs of N_ad_ did not overlap those of N_e_ (Table 5). Regarding the calculations of the N_ad_ values, the best-supported model using CAPWIRE, ECM, indicated that all individuals have an equal capture probability. In all cases, the *P* values of the LRT (i.e. likelihood ratio test) were well above 0.1 (*P*_females_ = 0.739; *P*_males_ = 0.334; *P*_total population_ = 0.644). By contrast, closed-capture model selection using MARK indicated best support for an increase in recapture probability over time (ΔAIC = 0.00; AIC Weight = 0.76; Supplementary Tables S1 and S2). Independent of the N_ad_ estimate used to calculate the adult population density, density was calculated to be 0.05 adult individuals/m². Lastly, the simple sex ratios of reproductively mature females and males were estimated to be 1:0.92 and 1:0.67 by CAPWIRE and MARK, respectively (Table 5).

**Table 5:** Estimates of potential breeding adults (N_ad_) in the Hula painted frog population under study as calculated by the programmes CAPWIRE and MARK. Abbreviation key: CI, confidence interval; n.a., not available; SE, standard error.

	Estimate	SE	CI		Software
			Lower	Upper	
N_ad_ (♀)	125	n.a.	95	163	
N_ad_ (♂)	115	n.a.	77	178	CAPWIRE
Total	236	n.a.	189	237	
N_ad_ (♀)	146	19	118	193	
N_ad_ (♂)	98	14	78	133	MARK
Total	244	32	196	325	

## Discussion

In this study, we used 18 newly developed polymorphic microsatellite loci, in addition to capture-recapture techniques based on individual recognition, to investigate the only known sizeable population of Hula painted frogs, which was recently discovered outside the protected boundaries of the Hula Nature Reserve. With exception of the nine Hula painted frog individuals we captured within the Hula Nature Reserve and included into the population structure analyses, all individuals analysed in this study were taken from this single spring-fed location.

Both effective population size estimates as well as estimates of potentially reproducing adults in the population appeared to be very low. By contrast, we found the population to exhibit a relatively high genetic diversity. Even though 89% ( = 155 individuals) of all Hula painted frogs that were captured after the species’ rediscovery were obtained from a single specific location (hereafter the ditch), the low and congruent N_ad_ estimates for this part of the population are a concern for its future survival. In particular, if considering that almost nothing is known on the maximum age or generation time of most amphibian species including Hula painted frogs. Therefore, we cannot exclude a bias in our results due to the possibility that we included aged and non-reproducing individuals in our calculation. While the adults of many other anurans only visit water bodies for reproduction purposes, we found that not only adult, but at least also some juvenile Hula painted frogs were dwelling in the water during the reproductive season. Consequently, our N_ad_ estimates possibly include breeders and senescent individuals. However, especially for the adult males encountered during our survey, we ascertained reproductive state by their well-expressed nuptial pads and good body condition. In addition, N_e_ estimates that we calculated with four methods with different underlying assumptions were likewise very low for this population, and may be equally alarming. When we compared our results to those of two other studies focusing on similar systems, i.e. geographically very restricted amphibian species without viable metapopulation structures, we found that comparatively low N_e_ values had also been reported (i) for the Black toad (*Anaxyrus exsul*, formerly *Bufo exsul*), a desert amphibian inhabiting four spring systems with a total range of only ~15 ha in California, for which N_e_ values ranged between 7 and 30^39^, and (ii) for the Montseny brook newt (*Calotriton arnoldi*), one of the most endangered European vertebrate species with an overall distribution range of only 8 km², for which N_e_ values ranged between 7.3 and 342.3^40^. These two species, however, already diverged from their sister species during the Pleistocene glaciations^39–41^ and successfully persisted until today despite their isolation. The habitat alterations that caused the decline of Hula painted frogs are far too recent, to allow a substantiated estimate regarding the relict population’s ability to persist with such low N_ad_ and N_e_ values.

Despite the fact that N_e_ values of < 100 have been found to be relatively common in amphibians^42–44^, and very low N_e_ values of < 50 have even been detected in populations of very common and widespread amphibian species, e.g. Common frog (*Rana temporaria*)^45^, Common toad (*Bufo bufo*)^46^, Crested newt (*Triturus cristatus*)^43^ and Natterjack toad (*Bufo calamita*)^47,48^, it has to be considered that all those investigated populations either exhibited comparatively low genetic variation^49^, low genetic diversity^47,48^ and/or the authors conceded that the populations under study may not have represented discrete populations as immigration from neighbouring populations could not be completely ruled out^43,45,46,49^. Fortunately, our data suggests that the Hula painted frog population under study seems to have yet maintained high allelic richness, high genetic diversity and a significantly negative inbreeding coefficient. Similar surprising findings, i.e. high allelic richness, high genetic diversity and a very low inbreeding coefficient, were also observed in the Black toad and the Montseny brook newt^39,40^. This demonstrates that species with small N_ad_ and N_e_ are not necessarily genetically depauperate. The explanations for this apparent contradiction are not easy to assess. For the Montseny brook newt, the authors speculate a possible preference of females to breed with (i) genetically more dissimilar partners or (ii) with several males in order to avoid low egg viability^40^. For the Hula painted frog, three further possible explanations may be speculated: (iii) recently metamorphosed (and potentially inbred) individuals could disperse^50^, which would give another explanation for the low number of juveniles observed by us, or (iv) there could be occasional immigrations of individuals from other yet unknown populations within the Hula Valley. However, even though previous observations suggest a low dispersal capability of this species^4^, and the habitat between the different water bodies cannot be considered amphibian-friendly so that an admixture between the different sites may be rather limited, occasional flooding events that occur every few years may have facilitated a dispersal of genetically divergent individuals from one location within the Hula Valley to another. Such sporadic immigrations could have led to an improved fitness of the investigated population; a phenomenon termed ‘genetic rescue’^51^. Last but not least, (v) we cannot rule out the possibility that, prior to the drainage, the whole Hula painted frog population might have been subdivided into several partly fixed subpopulations that were distributed around the former Lake Hula. In such a case, the reduction of the suitable habitat would have surely prompted a migration of individuals that finally gathered around the remaining permanent water bodies.

We found strong evidence that the individuals captured at the single location outside the Hula Nature Reserve (i.e. the ditch) belong to two genetically significantly different clusters. Therefore, the results of our population structural analyses seem to indeed support the last two hypotheses listed above, which are either genetic rescue from at least one unknown population nearby or a recent admixture of several partly fixed subpopulations that only recently congregated again. These two hypotheses are further supported by the high degree of linkage disequilibrium observed for the ditch population, while no linkage disequilibrium was detected for the nine individuals captured within the Hula Nature Reserve. Such high levels of linkage between unlinked markers are known to occur in admixed populations with high allele-frequency differences in the parental populations^52^.

After the drainage of the Lake Hula marshes in the 1950s, the former swamp area had turned into a dry, unsuitable habitat for amphibians that were most likely drawn to the last remaining aquiferous canals in the close vicinity of the former lake and marshes. Only in 1994, some parts of the drained peatland were re-flooded and shallow flood and drainage canals were created^53^, although most of them depend on sufficient rainfall. The investigated population could thus be an assortment of surviving individuals of two different subgroups for which the overall low N_e_ values would indicate a very poor recruitment success and concomitant admixture during recent years. We reject the idea of the alternative scenario, in which the drainage might have resulted in divided, tiny subpopulations affected strongly by genetic drift, as the time frame of 60–70 years after the drainage would be too short to have prompted such a strong effect, especially if a poor recruitment success is considered. It remains yet unclear which of the two explanations best describes reality. Recent environmental DNA (eDNA) analyses identified numerous other locations within the Hula Valley at which DNA traces of the Hula painted frog were found^54^, which suggests that genetic rescue may indeed be a plausible explanation. However, surprisingly, heterozygosities for each sub-cluster were relatively high. Expected and observed heterozygosities (mean ± SD) were 0.784 ± 0.073 and 0.796 ± 0.084 for sub-cluster 1, and 0.669 ± 0.113 and 0.727 ± 0.147 for sub-cluster 2. And also the allele frequencies differed markedly between the two sub-clusters (for details see Supplementary Table S3). Both findings, as well as the fact that the two sub-clusters were relatively large (sub-cluster 1: N = 89; sub-cluster 2: N = 45), may rather reflect the alternative scenario of recent admixture.

In any case, the current high genetic diversity of our main target population might have been favoured by the past drainage of the Hula marshes, by favouring contact among previously more isolated subpopulations. However, even though our combined results suggest at least one hidden Hula painted frog population nearby, we did not yet succeed in detecting another population.

The low population size estimates illustrate that the Hula painted frog may still be teetering on the brink of extinction, although it seems that it has still maintained an adequate genetic variance and that the drainage of the Hula Valley has had no negative effect on its evolutionary potential so far. We cannot yet directly relate our N_ad_ and N_e_ values, as demographic and genetic data collected during the same time period are not directly linked^25,55^. However, the low estimated values and the apparently poor reproductive success highlight the importance of immediate action in order to prevent further reduction of the species’ (effective) population size and guarantee its long-term survival.

We strongly advocate that immediate conservation actions should focus on a restoration project aiming at improving current habitat quality as well as maximising habitat connectivity for the Hula painted frog populations. First corridors between the protected Nature Reserve and the location of the focal population should be established without further delay in order to facilitate the migration of individuals between the two known locations and thus help maintain the genetic diversity of the species. Apart from habitat restoration, preservation and extension, future studies should urgently focus on detecting possible hidden populations of the Hula painted frog at those sites that have recently been identified by eDNA analyses. Furthermore, *in-situ* conservation measures such as translocation programmes, or *ex-situ* conservation measures such as captive breeding programmes should be considered, in order to increase the reproductive success of this species. Indeed, concerted efforts and integrative strategies will be essential as we come together to conserve this unique and rare amphibian.

## Methods

### Sample collection and processing

We captured a total of 125 Hula painted frog individuals at the focal population, a rather shallow but permanent ditch with a water volume of ~1000–1500 m^3^ near Yesod HaMa’ala^4^. We obtained buccal epithelial cells by rubbing the mucosa with a sterile cotton swab. Swabs were directly stored in 95–99% ethanol until further processing. For analyses regarding population structure and kinship inference, we further included the tissue of nine individuals that were captured within the Hula Nature Reserve^4^.

**Figure 3:**
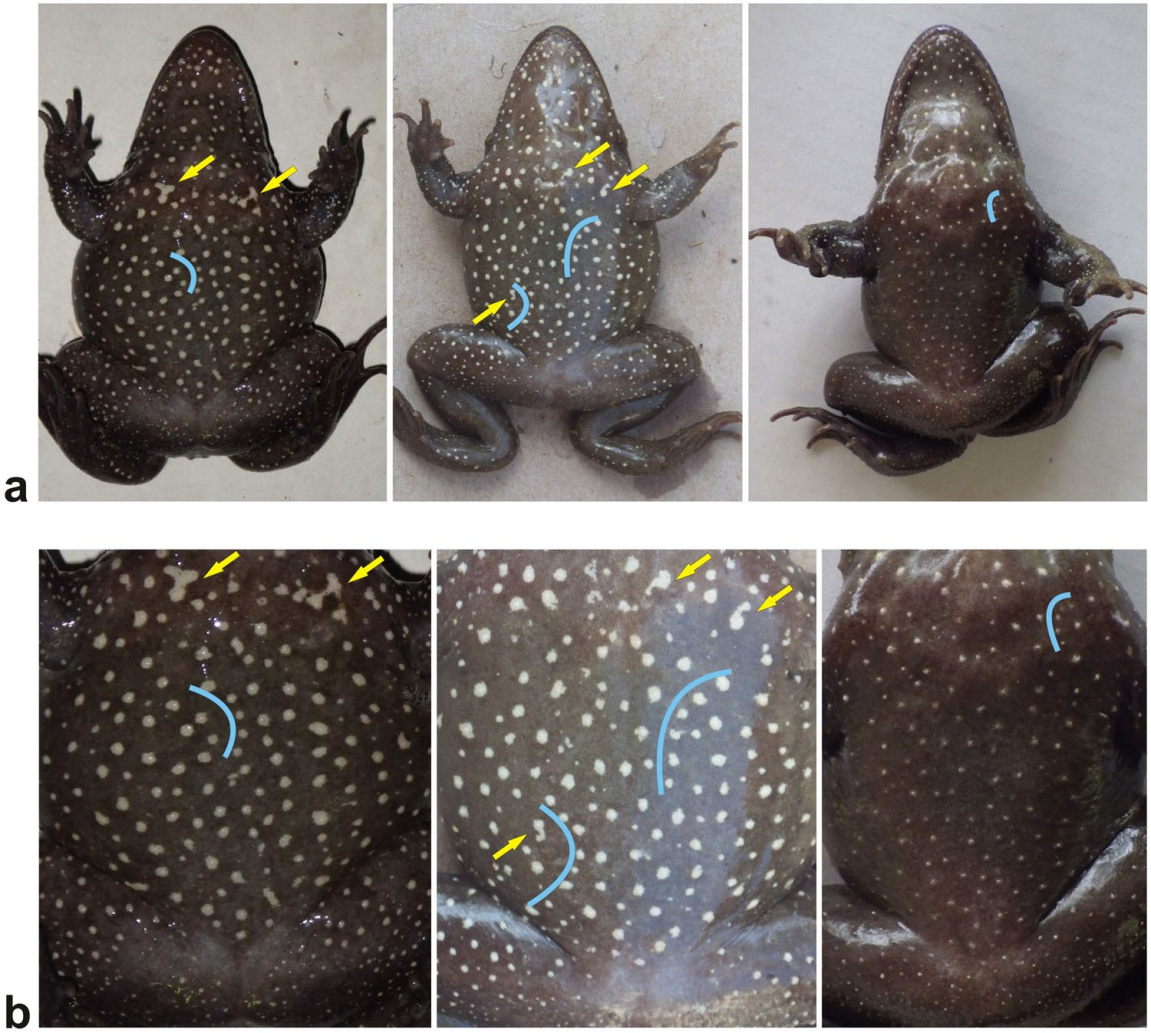
Digital photographs of the ventral spot pattern of three adult Hula painted frog individuals. (**a**) Example images of two adult female individuals with snout-vent lengths of 98.5 mm (left) and 88.0 mm (middle), and an adult male individual with a snout-vent length of 106.0 mm (right) as used by simple eye matching. The yellow arrows indicate examples of spots with a distinct shape and the blue lines indicate examples of characteristic strings of spots (to the right of the blue lines) that, together with the general appearance of the spot pattern, were used for the identification of recaptured individuals. (**b**) Cropped photographs of the same individuals (same order as above) as used for the automatic identification with Wild-ID. Photos by RGBP.

We used the distinctive natural ventral markings displayed by each metamorphosed frog for identifying re-caught individuals (Fig. 3). Our own observations seem to indicate that the spot pattern does not change substantially after the individuals reach one year of age (SVL of ~20–30 mm), and that it may be used to unambiguously identify juveniles with a SVL > 25 mm, and possibly smaller ones. As a reference for re-caught individuals, we constructed an electronic library, which included digital photographs of the ventral spot pattern of all captured individuals. Photographs of newly captured individuals were compared both by simple eye matching (Fig. 3a), and by automatic pattern identification software (Wild-ID version 1.0.0^56^; Fig. 3b) with the ones in our constructed database. The resulting capture-recapture data of adults was used to estimate the N_ad_ of this population. We calculated the N_ad_ rather than the N_b_ as our current knowledge on the species does not allow the calculation of the latter.

We extracted the genomic DNA using DNeasy Blood and Tissue Kits (Qiagen) following the manufacturer’s instructions with minor modifications: (i) elution buffer AE was heated in a water bath at 55 °C prior to use and (ii) elution buffer step was first performed with 70 µl buffer AE instead of 200 µl and then repeated with 30 µl of nuclease-free H_2_O.

We constructed a microsatellite library using DNA of three Hula painted frog individuals (vouchers: Am. 2572, Am. 2573 and Am. 2574), all collected within the Hula Valley in Israel) and the commercial service provided by the Sequencing Genotyping Facility, Cornell Life Sciences Core Laboratory Center (CLC), U.S.A. Out of this library, we chose 18 loci based on following criteria: (i) tetrameric, (ii) repeat motif between 10 and 15, (iii) less than 1000 reads, as deep coverage could indicate multiple copies and (iv) GC content of 50 (for details see Supplementary Table S4). All loci were tested for successful amplification and for yielding unambiguously scorable and polymorphic PCR products. We amplified the microsatellite loci following Schuelke^57^. Obtained PCR products were diluted with 15 µl of RNase-free water, and to 1 µl of each diluted product 15 µl of Genescan 500–ROX size standard (Applied Biosystems) were added. Fragment analysis was performed on a 3130xl Genetic Analyzer (Life Technologies). Allele scoring was done with GeneMapper^®^ (SoftGenetics, State College, PA, U.S.A) (for details see Supplementary Table S5).

Our initial analysis indicated departures from HWE for one locus and linkage between the majority of loci. However, our preliminary results indicate that for the nine individuals captured within the Hula Nature Reserve none appeared to be linked. We therefore conclude that the new microsatellite loci are highly informative and suitable for genetic variability as well as relatedness analyses, and also, in the future, for modelling connectivity between different populations of this rare species.

All methods were carried out in accordance with the guidelines and regulations regarding research on wild and protected animals in Israel. Sampling protocols were approved by the Israel Nature and Parks Authority that issues all permits for research work on wildlife in Israel (Permit numbers: 2013/39321 and 2015/40926).

### Genetic data analyses

To verify that each individual entered the analyses only once, we compared the genetic data of the previously visually assigned individuals with COLONY version 2.0.4.4^58^. Subsequently, we checked the loci of 125 Hula painted frog individuals for potential scoring errors and large allele dropout using MICRO-CHECKER version 2.2^59^. We tested for the presence of null alleles employing MICRO-CHECKER and GENEPOP version 4^60^. Tests for linkage disequilibrium between pairs of loci and deviations from Hardy-Weinberg equilibrium for each locus were carried out with GENEPOP version 4 and Arlequin 3.5.2.2^61^.

For each microsatellite locus, we calculated the mean number of alleles (A) and inbreeding coefficient (F_IS_)^62^ using FSTAT version 2.9.3.2.^63^, allelic diversity employing Shannon’s diversity index (^s^H)^64,65^ as implemented in GeneAlEx 6.503^66,67^ as well as observed heterozygosity (H_O_) and expected heterozygosity (H_E_) using Arlequin.

We inferred demographic history by calculating the N_e_ through four different approaches: (i) the maximum likelihood approach for sibship assignment as implemented in COLONY under the assumption of random mating, (ii) the one-sample linkage disequilibrium method^68^ and jackknife approach to estimate confidence intervals as implemented in LDNE version 1.31^69^ under the assumption of a minimum allele frequency of 2% to minimise potential bias caused by rare alleles^70^, (iii) the heterozygote excess method, and (iv) the molecular coanchestry method (NeEstimator v2.01)^71^.

In order to infer the population structure, we used the Bayesian clustering algorithm as implemented in STRUCTURE version 2.3.4^72^ under the assumption of an admixture model with correlated allele frequencies. We tested the number of clusters (*K*) from one to five considering a burn-in phase of 100,000 followed by 900,000 Markov Chain Monte Carlo (MCMC) iterations, and repeated each assessment of *K* ten times. We selected the optimal number of clusters following the Δ*K* method by Evanno *et al*.^73^ using STRUCTURE HARVESTER^74^.

Furthermore, we used the full likelihood (FL) method implemented in COLONY for assigning kinship from the individuals’ genotype data. COLONY allows the determination of female and male genotypes, giving it an advantage over other programs (e.g. ML-RELATE). We ran the analysis with high precision and with long run length. We further assumed polygamy for both sexes as well as possible inbreeding. All other parameters were kept default.

Moreover, we calculated Likelihood Relatedness (LR) among individuals using ML-RELATE^75^. In order to compare the results with those obtained by STRUCTURE, we performed a hierarchical clustering analysis on the ML-RELATE output. Therefore, we first calculated Euclidean distances and then applied the Ward’s criterion (ward.D2; 10,000 bootstraps) as implemented in R package ‘pvclust’^76,77^. Subsequently, we calculated the genetic differences (F_ST_) between the clusters obtained by STRUCTURE and ML-RELATE using Arlequin and FSTAT, thereby excluding the individuals for which both programs yielded deviating results. Furthermore, we verified the relationships among individuals as determined by COLONY with those obtained by ML-RELATE. Thereby, three different categories of relationships were considered by each program: half-siblings (HS), full-siblings (FS) and parent-offspring (PO). However, we decided to group the FS and PO estimates (FS/PO), as we have no data on the age of the individuals and cannot reliably support parent-offspring dyads.

Lastly, by analysing the COLONY-assigned family clusters that included captured juveniles, we tried to infer the numbers of successful breeding pairs between 2012 and 2014.

### Capture-recapture

We estimated the N_ad_ by using two approaches: one developed especially for genetic mark-recapture data and implemented in programme CAPWIRE^78^ and the closed population maximum-likelihood approach implemented in programme MARK^79,80^. CAPWIRE is based on a simple urn model which takes substantial capture heterogeneity into account and appears to yield reliable results, especially for smaller populations (N ≤ 100)^78,81,82^. It offers two different capture models: the even capture probability model (ECM) which assumes that each individual has the same probability of being captured in each sampling session, and the two innate rates model (TIRM), in which two types of individuals with unequal capture probabilities are assumed. Programme MARK offers over 100 different modules to estimate population parameters and by flexibly modelling recapture probability, allows estimation of the size of closed populations sampled over multiple occasions^83^. We restricted our dataset to the 118 captured adult individuals because we were interested in the breeding segment of the population. To determine which individuals were adults, we used a snout-vent length of >66.5 as a cut-off value^4^. Male and female recapture rates and population sizes were modelled separately.

Within CAPWIRE, models were run with minimum and maximum capturability ratios set at 1 and 20 and search increments for capturability ratio set at 0.1. We used a largest population size of 1000 for dimensioning and 95% confidence intervals for the estimate on population size based on 1000 bootstrap replicates. Following suggestions by Miller *et al*.^78^, we selected the best model based on the results of the implemented likelihood ratio test (LRT) that calculates and compares the log likelihood of each model and was set at *P* < 0.1 (default), i.e. if TIRM > ECM cannot not be rejected, ECM is accepted. As CAPWIRE does not allow simultaneous calculation of the N_ad_ estimates for females and males and the calculation of the N_ad_ estimate for the total population, we calculated the latter independently.

Within MARK, we used the implemented closed population capture-recapture approach. In order to avoid over-parameterisation, we pooled each set of consecutive capture nights into one capture occasion. For each individual, we built a capture history based on detections for every given occasion. We used the full likelihood closed-capture module^84^ to build several competing models, including temporal and behavioural effects. We initially modelled capture probability (p) and recapture probability (c) separately to examine a possible detection response. Because our ability to detect and capture Hula painted frog individuals improved over time, we built models allowing for capture rates to differ among capture occasions and periods. We selected the model based on Akaike’s Information Criterion values (AIC)^85^, examined model convergence using real and derived parameter estimates^80^, and used calculations performed by MARK to assess support as suggested by relative AIC_c_ weights. We derived point density estimates for N_ad_ and 95% confidence intervals (CIs) using the top-ranked model.

Lastly, adult population densities for the location under study were estimated by dividing each N_ad_ estimate by the area of the ditch (5,060 m²). Even though such local assessments of amphibian densities rarely exist, and comparisons to other amphibian populations are thus not feasible, this estimate may aid us in monitoring the focal population and possibly, in the future, in comparing it to the density estimates obtained for other Hula painted frog populations.

### Data Availability

All data generated or analysed during the current study are included in this published article (and its Supplementary Information files).

## Electronic supplementary material

Supplementary Information

## References

[1] Honegger R (1981). List of amphibians and reptiles either known or thought to have become extinct since 1600. Biol. Conserv..

[2] Dimentman, C. H., Bromley, H. J. & Por, F. D. *Lake Hula: Reconstruction of the Fauna and Hydrobiology of a Lost Lake*. (The Israel Academy of Sciences and Humanities, Jerusalem, 1992).

[3] Biton R (2013). The rediscovered Hula painted frog is a living fossil. Nat. Commun..

[4] Perl, R. G. B. *et al*. Natural history and conservation of the rediscovered Hula painted frog, *Latonia nigriventer. Contrib. Zool.* **86**, 11–37 (2017).

[5] IUCN. *The IUCN Red List of Threatened Species. Version2017–2*, http://www.iucnredlist.org (2017). (Date of access: 02.10.2017)

[6] Minton SA (1968). The fate of amphibians and reptiles in a suburban area. J. Herpet..

[7] Terborgh J (1974). Preservation of natural diversity: the problem of extinction prone species. BioScience.

[8] Bradshaw AD (1977). Conservation problems in the future. Proc. R. Soc. Lond. B.

[9] Ehrlich PR (1982). Human carrying capacity, extinctions, and nature reserves. BioScience.

[10] Wilcox, B. A. & Murphy, D. D. Conservation strategy: the effects of fragmentation on extinction. *Am. Nat.* **125**, 879–887 (1985).

[11] Stuart SN (2004). Status and trends of amphibian declines and extinctions worldwide. Science.

[12] Fischer J, Lindenmayer DB (2007). Landscape modification and habitat fragmentation: a synthesis. Glob. Ecol. Biogeogr..

[13] Shaffer ML (1981). Minimum population sizes for species conservation. BioScience.

[14] Gibbs JP (2001). Demography versus habitat fragmentation as determinants of genetic variation in wild populations. Biol. Conserv..

[15] Keyghobadi N, Roland J, Matter SF, Strobeck C (2005). Among- and within-patch components of genetic diversity respond at different rates to habitat fragmentation: an empirical demonstration. Proc. R. Soc. Lond. B.

[16] Soulé, M. E. & Wilcox, B. A. *Conservation Biology. An Evolutionary-Ecological Perspective*. (Sinauer, Sunderland, 1980).

[17] Mills L, Smouse P (1994). Demographic consequences of inbreeding in remnant populations. Am. Nat..

[18] Keller LF, Waller DM (2002). Inbreeding effects in wild populations. Trends Ecol. Evol..

[19] Lande R (1988). Genetics and demography in biological conservation. Science.

[20] Leigh EG (1981). The average lifetime of a population in a varying environment. J. Theoret. Biology.

[21] Frankel, O. & Soulé, M. E. *Conservation and Evolution*. (Cambridge University Press, Cambridge, 1981).

[22] Murphy, D. D., Freas, K. E. & Weiss, S. B. An environment‐metapopulation approach to population viability analysis for a threatened invertebrate. *Conserv. Biol.* **4**, 41–51 (1990).

[23] Allentoft M, O’Brien J (2010). Global amphibian declines, loss of genetic diversity and fitness: a review. Diversity.

[24] Rodriguez de Cara MA, Villanueva B, Toro MA, Fernandez J (2013). Using genomic tools to maintain diversity and fitness in conservation programmes. Mol. Ecol..

[25] Palstra, F. P. & Fraser, D. J. Effective/census population size ratio estimation: a compendium and appraisal. *Ecol. Evol.* **2**, 2357–2365 (2012). 10.1002/ece3.329 PMC3488685 23139893

[26] Álvarez D, Lourenço A, Oro D, Velo-Antón G (2015). Assessment of census (N) and effective population size (N_e_) reveals consistency of N_e_ single-sample estimators and a high N_e_/N ratio in an urban and isolated population of fire salamanders. Conserv. Genet. Resour..

[27] Miller CR, Waits LP (2003). The history of effective population size and genetic diversity in the Yellowstone grizzly (*Ursus arctos*): implications for conservation. Proc. Natl. Acad. Sci. USA.

[28] Saura M, Caballero P, Caballero A, Morán P (2006). Genetic variation in restored Atlantic salmon (*Salmo salar* L.) populations in the Ulla and Lérez rivers, Galicia, Spain. ICES J. Mar. Sci..

[29] Marshall JC, Kingsbury BA, Minchella DJ (2009). Microsatellite variation, population structure, and bottlenecks in the threatened copperbelly water snake. Conserv. Genet..

[30] Bateson ZW (2014). Genetic restoration of a threatened population of greater prairie-chickens. Biol. Conserv..

[31] Finger AJ, May B (2015). Conservation genetics of a desert fish species: the Lahontan tui chub (*Siphateles bicolor* ssp.). Conserv. Genet..

[32] Estoup A (1998). Comparative analysis of microsatellite and allozyme markers: a case study investigating microgeographic differentiation in brown trout (*Salmo trutta*). Mol. Ecol..

[33] Broquet T, Berset-Braendli L, Emaresi G, Fumagalli L (2007). Buccal swabs allow efficient and reliable microsatellite genotyping in amphibians. Conserv. Genet..

[34] Petersen CGJ (1896). The yearly immigration of young plaice into the Limfjord from the German Sea. Rep. Danish Biol. Stat..

[35] Williams, B. K., Nichols, J. D. & Conroy, M. J. C. *Analysis and Management of Animal Populations*. (Academic Press, San Diego, 2002).

[36] Slatkin M (1985). Gene flow in natural populations. Ann. Rev. Evol. Syst..

[37] Adams BK, Hutchings JA (2003). Microgeographic population structure of brook charr: a comparison of microsatellite and mark‐recapture data. J. Fish Biol..

[38] Lowe WH, Allendorf FW (2010). What can genetics tell us about population connectivity?. Mol. Ecol..

[39] Wang IJ (2009). Fine-scale population structure in a desert amphibian: landscape genetics of the black toad (*Bufo exsul*). Mol. Ecol..

[40] Valbuena-Ureña E, Soler-Membrives A, Steinfartz S, Orozco-terWengel P, Carranza S (2017). No signs of inbreeding despite long-term isolation and habitat fragmentation in the critically endangered Montseny brook newt (*Calotriton arnoldi*). Heredity.

[41] Goebel AM, Ranker TA, Corn PS, Olmstead RG (2009). Mitochondrial DNA evolution in the *Anaxyrus boreas* species group. Mol. Phylogenet. Evol..

[42] Funk WC, Tallmon DA, Allendorf FW (1999). Small effective population size in the long-toed salamander. Mol. Ecol..

[43] Jehle R, Arntzen JW, Burke T, Krupa AP, Hödl W (2001). The annual number of breeding adults and the effective population size of syntopic newts (*Triturus cristatus*, *T. marmoratus*). Mol. Ecol..

[44] Beebee TJ, Griffiths RA (2005). The amphibian decline crisis: a watershed for conservation biology?. Biol. Conserv..

[45] Schmeller DS, Merilä J (2007). Demographic and genetic estimates of effective population and breeding size in the amphibian *Rana temporaria*. Conserv. Biol..

[46] Scribner KT, Arntzen JW, Burke T (1997). Effective number of breeding adults in *Bufo bufo* estimated from age‐specific variation at minisatellite loci. Mol. Ecol..

[47] Rowe, G. Microsatellite heterozygosity, fitness, and demography in natterjack toads *Bufo calamita. Anim. Conserv.* **2**, 85–92 (1999).

[48] Rowe G, Beebee TJC (2004). Reconciling genetic and demographic estimators of effective population size in the anuran amphibian *Bufo calamita*. Conserv. Genet..

[49] Rowe G, Beebee TJ (2003). Population on the verge of a mutational meltdown? Fitness costs of genetic load for an amphibian in the wild. Evolution.

[50] Blouin SF, Blouin M (1988). Inbreeding avoidance behaviours. Trends Ecol. Evol..

[51] Ingvarsson PK (2001). Restoration of genetic variation lost – the genetic rescue hypothesis. Trends Ecol. Evol..

[52] Pfaff, C. L. *et al*. Population structure in admixed populations: effect of admixture dynamics on the pattern of linkage disequilibrium. *Am. J. Hum. Genet.* **68**, 198–207 (2001). 10.1086/316935 PMC1234913 11112661

[53] Hambright, K. D. & Zohary, T. The Hula Valley (northern Israel) wetlands rehabilitation project in *An International Perspective on Wetland Rehabilitation* (ed. Streever, W.) 173–180 (Springer Science + Business Media, Dordrecht, 1999).

[54] Renan, S. *et al*. Living quarters of a living fossil – uncovering the current distribution pattern of the rediscovered Hula painted frog (*Latonia nigriventer*) using environmental DNA. *Mol. Ecol.* **26**, 6801–6812 (2017). 10.1111/mec.14420 29117632

[55] Nunney L (1995). Measuring the ratio of effective population size to adult numbers using genetic and ecological data. Evolution.

[56] Bolger DT, Morrison TA, Vance B, Lee D, Farid H (2012). A computer-assisted system for photographic mark-recapture analysis. Methods Ecol. Evol..

[57] Schuelke M (2000). An economic method for the fluorescent labeling of PCR fragments. Nat. Biotechnol..

[58] Jones OR, Wang J (2010). COLONY: a program for parentage and sibship inference from multilocus genotype data. Mol. Ecol. Resour..

[59] Van Oosterhout C, Hutchinson WF, Wills DPM, Shipley P (2004). MICRO-CHECKER: software for identifying and correcting genotyping errors in microsatellite data. Mol. Ecol. Notes.

[60] Rousset F (2008). GENEPOP'007: a complete re-implementation of the GENEPOP software for Windows and Linux. Mol. Ecol. Resour..

[61] Excoffier, L. & Lischer, H. E. L. Arlequin suite ver 3.5: a new series of programs to perform population genetics analyses under Linux and Windows. *Mol. Ecol. Resour.* **10**, 564–567 (2010). 10.1111/j.1755-0998.2010.02847.x 21565059

[62] Weir BS, Cockerham CC (1984). Estimating F-statistics for the analysis of population structure. Evolution.

[63] Goudet J (1995). FSTAT (version 1.2): a computer program to calculate F-statistics. J. Hered..

[64] Shannon CE (1948). A mathematical theory of communication. Bell Syst. Tech. J..

[65] Sherwin, W. B. Entropy and information approaches to genetic diversity and its expression: genomic geography. *Entropy* **12**, 1765–1798 (2010).

[66] Peakall R, Smouse PE (2006). GENALEX 6: genetic analysis in Excel. Population genetic software for teaching and research. Mol. Ecol. Notes.

[67] Peakall R, Smouse PE (2012). GenAlEx 6.5: genetic analysis in Excel. Population genetic software for teaching and research – an update. Bioinformatics.

[68] Hill WG (1981). Estimation of effective population size from data on linkage disequilibrium. Genet. Res..

[69] Waples RS, Do C (2008). LDNE: a program for estimating effective population size from data on linkage disequilibrium. Mol. Ecol. Resour..

[70] Waples RS, Do C (2010). Linkage disequilibrium estimates of contemporary N_e_ using highly variable genetic markers: a largely untapped resource for applied conservation and evolution. Evol. Appl..

[71] Do, C. *et al*. NeEstimatorv2: re-implementation of software for the estimation of contemporary effective population size (N_e_) from genetic data. *Mol. Ecol. Resour.* **14**, 209–214 (2014). 10.1111/1755-0998.12157 23992227

[72] Pritchard JK, Stephens M, Donnelly P (2000). Inference of population structure using multilocus genotype data. Genetics.

[73] Evanno G, Regnaut S, Goudet J (2005). Detecting the number of clusters of individuals using the software STRUCTURE: a simulation study. Mol. Ecol..

[74] Earl, D. A. & vonHoldt, B. M. STRUCTURE HARVESTER: a website and program for visualizing STRUCTURE output and implementing the Evanno method. *Conserv. Genet. Resour*. **4**, 359–361 (2012).

[75] Kalinowski, S. T., Wagner, A. P. & Taper, M. L. ML-RELATE: a computer program for maximum likelihood estimation of relatedness and relationship. *Mol. Ecol. Notes* **6**, 576–579 (2006).

[76] R Development Core Team. R: a language and environment for statistical computing. R Foundation for Statistical Computing, Vienna, Austria. http://www.R-project.org/ (2013).

[77] Suzuki R, Shimodaira H (2006). Pvclust: an R package for assessing the uncertainty in hierarchical clustering. Bioinformatics.

[78] Miller, C. R., Joyce, P. & Waits, L. P. A new method for estimating the size of small populations from genetic mark–recapture data. *Mol. Ecol.* **14**, 1991–2005 (2005). 10.1111/j.1365-294X.2005.02577.x 15910321

[79] White GC, Burnham KP (1999). Program MARK: survival estimation from populations of marked animals. Bird Stud..

[80] Cooch, E. G. & White, G. (eds). *Program MARK – A Gentle Introduction*. http://www.phidot.org/software/mark/docs/book/ (Date of access: 08.09.2017) (2014).

[81] Arrendal J, Vila C, Björklund M (2007). Reliability of noninvasive genetic census of otters compared to field censuses. Conserv. Genet..

[82] Robinson, S. J., Waits, L. P. & Martin, I. D. Estimating abundance of American black bears using DNA-based capture–mark–recapture models. *Ursus* **20**, 1–11 (2009).

[83] Luikart G, Ryman N, Tallmon DA, Schwartz MK, Allendorf FW (2010). Estimation of census and effective population sizes: the increasing usefulness of DNA-based approaches. Conserv. Genet..

[84] Pledger S (2000). Unified maximum likelihood estimates for closed capture–recapture models using mixtures. Biometrics.

[85] Burnham, K. & Anderson, A. *Model Selection and Multimodel Inference: A Practical Information-Theoretic Approach*. (Springer-Verlag, Berlin, 2002).

