# Supplementary material for: Population genetic analysis of the recently rediscovered Hula painted frog (Latonia nigriventer) reveals high genetic diversity and low inbreeding
Source: Sci Rep. 2018 Apr 3;8:5588. doi: 10.1038/s41598-018-23587-w (PMC5882862; doi:10.1038/s41598-018-23587-w)
Supplement: Supplementary file 1 — Supplementary Information [file 41598_2018_23587_MOESM1_ESM.pdf]

Supplementary Information

Population genetic analysis of the recently rediscovered Hula painted frog (*Latonia nigriventer*) reveals high genetic diversity and low inbreeding

R. G. Bina Perl, Eli Geffen, Yoram Malka, Adi Barocas, Sharon Renan, Miguel Vences, Sarig Gafny

Supplementary Table S1. Comparison of the two CAPWIRE models ECM (even capture probability model) and TIRM (two innate rates model) as calculated for each sex and the total population (♀+♂). In the model selection process, ECM was used unless rejected using LRT (*P* value < 0.1). Abbreviation key: SS, sample size; Tobs, number of distinct individuals observed in sample; N^, CAPWIRE estimate of population size; LowCI, lower confidence bound; UpCI, upper confidence bound; CIWidth, upCI – lowCI; Model, model used a priori to analyse the data; L(ECM), best ln likelihood score under ECM; L(TIRM), best ln likelihood score under TIRM; Lambda, L(TIRM) - L(ECM); p(Lambda), proportions of simulations (under the ECM model) where observed ratio larger than observed value (= *P* value); Na^, point estimate of the number of type A individuals in population; Nb^, point estimate of the number of type B individuals in population; alpha^, point estimate of relative capture probability of type A individuals to type B individuals; Avg#Obs/Ind, average number of times sampled individuals were observed; CaptureCutOff, number of captures at or below which an individual is assigned as type B (individuals captured more often are viewed as type A individuals with relative capture probability alpha^).

Population	SS	Tobs	N^	LowCI	UpCI	CIWidth	Model	L(ECM)	L(TIRM)	Lambda	p(Lambda)	Na^	Nb^	alpha^	Avg#Obs/Ind	CaptureCutOff
♀	102	70	125	95	163	68	ECM	-409.381511	-394.4313144	14.9502	0.739	26	127	4.36014	1.457143	1
♂	67	51	115	82	178	96	ECM	-241.5289207	-230.6514699	10.87745	0.334	13	130	4.989879	1.313725	1
♀+♂	169	121	236	189	296	107	ECM	-762.839564	-736.6183341	26.22123	0.644	39	252	4.570356	1.396694	1

Supplementary Table S2. Comparison of seven MARK models fitted to capture-recapture data collected from the Hula painted frog population under study. We selected the best model (asterisked) based on its Akaike's Information Criterion values (AIC) (see text for details). Abbreviation key: AIC_c, AIC value corrected for small sample sizes; ΔAIC_c, difference in AIC_c between focal and best model.

Model	AIC_c	ΔAIC_c	AIC_c Weights	Model Likelihood	Number of Parameters	Deviance
{p=c(1=3=4=8,2,5=6=9, 7)}*	-0.51	0.00	0.76	1.00	6	132.34
{p=c(1=3=4=7=8,2,5=6=9)}	1.95	2.47	0.22	0.29	5	136.83
{p=c(t)}	7.38	7.89	0.01	0.02	11	130.08
{p=c(1=3=4, 2, 5=6=7=8)}	11.36	11.87	0.00	0.00	5	146.23
{p=c(1:4, 5:9)}	12.12	12.63	0.00	0.00	4	149.01
{p(.)=c(.)}	52.76	53.27	0.00	0.00	3	191.67
{p=c(sex)}	54.00	54.52	0.00	0.00	4	190.90

Supplementary Table S3. Genetic parameter estimates for 18 microsatellite loci for the two sub-clusters of the Hula painted frog population under study. Calculations are based on the two sub-clusters as obtained by STRUCTURE analysis. N, sample size; A, number of alleles per locus; ^{s}H, Shannon's diversity index; H_O, observed heterozygosity; H_E, expected heterozygosity; F_{IS}, inbreeding coefficient.

	Sub-cluster 1						Sub-cluster 2					
Locus	N	A	^{s}H	H_O	H_E	F_{IS}	N	A	^{s}H	H_O	H_E	F_{IS}
LAT1	89	10.000	1.606	0.832	0.761	-0.092	45	5.000	1.455	0.733	0.662	-0.109
LAT2	86	12.000	2.143	0.837	0.875	0.059	45	7.000	1.975	0.778	0.693	-0.123
LAT3	89	7.000	1.086	0.663	0.636	-0.031	45	4.000	1.202	0.422	0.355	-0.191
LAT4	89	7.000	1.830	0.888	0.825	-0.076	45	6.000	1.795	0.822	0.669	-0.232
LAT5	89	8.000	1.372	0.685	0.705	0.038	45	3.000	1.294	0.578	0.557	-0.037
LAT6	87	11.000	2.137	0.885	0.880	-0.012	45	8.000	2.205	0.956	0.853	-0.122
LAT7	89	8.000	1.732	0.742	0.774	0.045	45	5.000	1.647	0.978	0.774	-0.267
LAT8	89	13.000	2.173	0.910	0.875	-0.035	45	9.000	2.320	0.867	0.816	-0.063
LAT9	89	9.000	1.672	0.831	0.806	-0.019	45	5.000	1.635	0.733	0.655	-0.121
LAT10	86	7.000	1.541	0.791	0.742	-0.043	45	5.000	1.517	0.778	0.698	-0.115
LAT11	87	6.000	1.211	0.621	0.655	0.057	45	3.000	1.288	0.622	0.593	-0.051
LAT12	85	8.000	1.779	0.847	0.829	-0.026	44	6.000	1.782	0.545	0.592	0.080
LAT13	89	11.000	1.850	0.809	0.836	0.052	45	8.000	1.770	0.600	0.668	0.103
LAT14	89	6.000	1.431	0.764	0.756	-0.051	45	4.000	1.451	0.600	0.578	-0.038
LAT17	89	9.000	1.882	0.831	0.826	-0.002	45	6.000	1.927	0.778	0.737	-0.056
LAT18	85	12.000	1.842	0.753	0.744	-0.026	41	6.000	1.623	0.643	0.657	0.022
LAT19	86	9.000	1.568	0.732	0.777	0.047	41	5.000	1.712	0.805	0.722	-0.117
LAT20	89	10.000	1.716	0.910	0.856	-0.075	44	6.000	1.703	0.841	0.762	-0.104
Mean	88	9.056	1.698	0.796	0.787	-0.011	44	5.611	1.683	0.727	0.669	-0.086

Supplementary Table S4. Characterisation of the full set of 18 newly developed microsatellite loci for the Hula painted frog. Locus name, primer name (F = forward, R = reverse), repeat motif, number of alleles per locus (N_A) and amplified fragment size range (bp) are given for each locus. Forward primers were modified with a universal M13(-21) tail (18bp; TGTAAAACGACGGCCAGT) at the 5'-end. Annealing temperature was set at 53 °C for all primer pairs.

Locus name	Primer name	Primer sequence (5' 3')	Repeat motif	N_A	Size range (bp)
LAT1	LAT_F1	CAGGAAGATTTCATAGGCAGGC	AGAT(14)	6	409–445
	LAT_R1	GAGATTGGAGGAGGTTAGGAGC			
LAT2	LAT_F2	TATACTGGACGGCATCTTCAGG	AGAT(15)	10	275–323
	LAT_R2	TTCTAGCTCCACCACTGAACAG			
LAT3	LAT_F3	GGAAGACTGCTCCAATAGTTGC	AGAT(11)	7	238–270
	LAT_R3	GTCACATTACGTTTCGAGGAGG			
LAT4	LAT_F4	CATAGATGGAAGTGTGGCGTTC	AGAT(13)	7	299–327
	LAT_R4	CAGTTACTGCTTAGACCACAGC			
LAT5	LAT_F5	GGTCCTGTGCATGAGATTGAAG	AGAT(12)	6	252–296
	LAT_R5	ATGCTCTTCCTATTCTGCCCTC			
LAT6	LAT_F6	ATCTGGAATCTGGTCTACTGGC	AGAT(14)	10	257–317
	LAT_R6	GTAATAACGGACCCACAGTGAG			
LAT7	LAT_F7	GAAGGACTTTATCTGCACTGCC	AGAT(10)	7	322–346
	LAT_R7	CGGAACTGTCGGCAATAAGTAG			
LAT8	LAT_F8	CAATTAGCCTCTGATCACTGCC	AGAT(14)	12	243–299
	LAT_R8	ATCTGGTGCTACTCATGCTACC			
LAT9	LAT_F9	GTTATGCGCCATACCAGACTAC	AGAT(13)	9	244–276
	LAT_R9	GGTCATAGCAGAGAAGTCAGAC			
LAT10	LAT_F10	GCAATGTGAACCTTACTACGGC	AGAT(10)	6	239–263
	LAT_R10	TGCTTATGACGAGTGGGATAGG			
LAT11	LAT_F11	TCTAGCCATCACCAGGAGTTAG	AGAT(15)	5	278–294
	LAT_R11	ATCCTGTGGTACTGATCCTGTG			
LAT12	LAT_F12	ATACTAGACCTGACATCCTCCC	AGAT(14)	9	371–403
	LAT_R12	TCTGTTCATCTTCAGCTCCTCC			
LAT13	LAT_F13	CAAAGAGTCATACCACCAGAGG	AGAT(10)	9	246–290
	LAT_R13	GCATGGTGTAGCATAGTCTGTC			
LAT14	LAT_F14	GCCAGGAATTCAGACTAAGTGC	AGAT(10)	6	224–248
	LAT_R14	CTTCTCCACTTCATTGCCAAGG			
LAT17	LAT_F17	TCACAGGGTCATAGAGAGTTGG	AGAT(14)	9	228–268
	LAT_R17	ACTGACTTCCACCACATAGAGG			
LAT18	LAT_F18	AGAGGGTCTGCATTACGGTAAC	AGAT(11)	11	276–316
	LAT_R18	GTTAGTTGGATGCCGGATAACC			
LAT19	LAT_F19	GGTCATCTGGTGCATAAAGGTC	AGAT(10)	9	266–294
	LAT_R19	TACCACAGATCAACTCCTCCTG			
LAT20	LAT_F20	CATTGTCCTCTTCAGAAGCCAC	AGAT(11)	8	292–332
	LAT_R20	GTGTTCTCGCTTATCTTCACCC			

Supplementary Table S5. Allele profiles of 134 Hula painted frog individuals at 18 microsatellite loci. OPR, outside protected nature reserve; HNR, Hula Nature Reserve.

Individual ID	Location	LAT1	LAT2	LAT3	LAT4	LAT5	LAT6	LAT7	LAT8	LAT9	LAT10	LAT11	LAT12	LAT13	LAT14	LAT17	LAT18	LAT19	LAT20
L#6	HNR	425:449	295:307	238:250	307:319	288:296	253:257	330:346	271:299	256:272	247:255	294:294	375:383	258:286	228:236	228:268	304:308	266:270	308:316
L#7	HNR	449:453	279:307	238:258	327:327	252:288	253:277	330:338	267:275	256:260	243:243	278:286	375:387	266:270	232:232	236:236	288:292	266:290	296:296
L#9	HNR	429:433	283:299	246:246	327:327	264:296	261:273	326:334	275:283	252:264	243:243	286:294	395:395	266:286	236:240	236:268	288:304	266:278	300:328
L#14	HNR	421:453	279:315	238:246	323:327	264:272	261:317	338:346	279:291	244:256	243:243	286:290	387:403	270:270	224:232	248:248	280:288	266:274	296:296
L#15	HNR	429:457	279:307	238:258	315:315	252:288	257:257	330:342	255:283	248:252	243:247	290:302	383:391	250:274	232:232	228:244	304:312	262:270	296:300
L#16	HNR	425:449	287:315	238:238	315:327	268:292	257:281	302:334	271:283	248:256	239:239	290:294	383:395	270:270	224:224	248:248	304:308	282:290	300:308
L#17	OPR	425:433	283:299	238:258	315:319	276:276	257:301	338:338	283:283	252:268	251:251	286:286	379:379	254:266	228:228	244:252	280:304	266:266	328:328
L#18	OPR	425:429	279:295	246:270	323:323	272:296	277:281	322:334	259:267	252:260	243:251	286:290	383:391	262:278	228:248	260:260	284:296	266:274	312:312
L#19	OPR	425:433	295:319	246:246	299:311	264:296	273:301	326:338	267:283	248:252	243:251	286:286	391:403	266:270	228:240	260:268	288:288	266:270	312:320
L#20	OPR	425:433	279:295	238:246	299:327	264:272	257:301	326:334	283:295	248:256	239:243	290:290	391:403	254:270	224:240	260:268	288:312	266:270	312:320
L#21	OPR	429:433	275:279	246:246	323:327	264:272	269:293	334:334	283:295	256:260	239:243	286:290	375:391	270:286	224:240	260:268	288:296	270:270	300:312
L#22	OPR	429:433	279:295	246:246	299:327	272:296	257:269	326:326	259:295	248:252	251:251	286:290	391:403	254:270	228:228	228:248	288:312	266:270	320:332
L#23	OPR	425:429	275:295	246:246	311:327	264:296	273:281	326:346	259:271	248:252	243:251	286:290	391:391	266:266	228:240	260:268	288:288	266:266	296:324
L#24	OPR	433:433	299:319	238:246	319:327	264:264	269:277	326:334	267:295	252:256	239:251	286:294	391:391	262:286	224:228	252:260	304:308	274:274	328:332
L#25	OPR	429:445	295:319	246:246	323:327	264:264	261:269	322:334	267:271	252:260	243:259	282:286	391:399	254:266	240:240	260:260	284:300	266:274	300:328
L#26	OPR	425:433	295:299	246:246	311:315	264:296	257:269	322:338	283:283	260:276	243:251	290:294	391:395	266:286	228:236	260:268	288:304	266:294	292:328
L#27	OPR	425:429	319:319	246:246	311:323	264:296	269:293	322:326	259:259	252:252	243:243	286:290	391:391	266:274	240:240	236:236	280:288	266:270	312:328
L#28	OPR	429:433	299:319	238:246	299:311	264:296	261:273	322:338	259:259	252:252	243:259	286:290	391:391	278:278	240:240	236:236	292:292	266:270	312:324
L#29	OPR	429:445	279:283	246:246	323:323	264:296	261:293	338:338	243:271	252:256	243:255	282:290	391:391	266:270	232:240	260:260	280:288	266:274	324:324
L#30	OPR	425:433	295:319	238:246	323:323	264:264	269:273	322:338	259:283	252:252	243:259	286:290	391:391	266:278	240:240	236:260	288:292	266:270	324:328
L#31	OPR	425:429	319:319	238:246	299:311	296:296	261:293	322:326	259:259	252:252	243:259	286:286	391:391	278:278	240:240	236:236	280:292	266:270	312:324
L#32	OPR	429:433	275:283	246:254	311:323	276:296	273:273	322:338	243:291	248:252	243:251	286:294	379:391	254:266	224:228	232:244	288:288	294:294	312:312
L#33	OPR	433:433	299:299	238:246	319:327	264:264	277:281	326:334	259:295	252:252	239:251	286:286	391:391	262:286	224:228	252:260	288:304	270:274	300:320
L#34	OPR	425:429	319:319	238:246	299:323	264:296	261:273	322:326	259:283	252:252	243:259	286:286	391:391	266:274	240:240	236:236	292:292	266:266	324:328
L#35	OPR	429:429	319:319	246:246	323:327	264:296	257:293	322:326	283:283	252:252	243:255	282:286	391:391	270:278	232:240	232:268	280:292	278:294	312:328
L#36	OPR	425:433	283:323	238:270	315:315	264:276	269:273	326:342	283:283	252:264	243:251	290:294	383:395	254:266	228:248	248:252	304:304	266:274	292:320
L#37	OPR	433:445	283:295	246:246	323:327	264:264	257:261	322:338	271:287	256:260	243:259	282:290	391:395	266:266	240:240	232:260	284:288	274:294	300:324
L#38	OPR	425:433	295:319	246:246	307:315	264:264	257:261	326:326	259:283	248:264	243:251	286:290	395:399	266:266	228:236	244:248	288:288	274:274	296:300
L#39	OPR	425:429	283:319	246:246	307:327	264:264	269:277	334:334	267:279	252:256	251:263	286:286	399:403	270:278	228:228	236:248	000:000	000:000	320:328
L#40	HNR	421:449	279:299	238:254	315:323	288:288	257:269	302:338	271:291	244:248	243:255	278:290	383:403	254:270	236:236	244:244	312:324	290:294	296:300
L#41	HNR	433:433	275:279	238:246	307:319	264:296	269:281	334:338	267:295	256:264	243:243	282:290	383:387	262:266	232:232	252:256	000:000	000:000	292:292
L#42	OPR	425:425	279:295	246:270	315:323	296:296	269:277	322:346	259:267	248:260	243:243	286:286	383:391	266:278	228:240	236:260	288:292	266:274	312:312

Supplementary Table S5. Continued.

Individual ID	Location	LAT1	LAT2	LAT3	LAT4	LAT5	LAT6	LAT7	LAT8	LAT9	LAT10	LAT11	LAT12	LAT13	LAT14	LAT17	LAT18	LAT19	LAT20
L#43	OPR	425:429	279:319	246:246	323:327	296:296	257:293	326:334	243:259	248:252	243:251	286:290	391:391	270:286	228:240	260:260	000:000	000:000	312:328
L#44	HNR	425:437	279:307	238:258	315:315	276:276	261:277	326:338	267:267	252:256	255:259	286:294	379:387	258:270	228:232	236:244	280:316	266:266	296:300
L#45	OPR	429:429	275:283	238:246	307:319	276:276	000:000	322:322	267:283	260:264	243:251	286:286	000:000	266:278	228:228	232:260	280:316	266:266	292:292
L#46	OPR	433:433	303:303	246:258	307:315	264:264	257:257	342:346	267:283	256:256	243:251	286:286	391:395	266:286	228:240	252:252	000:000	266:274	320:328
L#47	OPR	409:429	303:319	246:254	327:327	264:264	281:301	326:334	267:295	252:260	239:251	282:290	395:395	254:266	224:228	244:260	288:300	266:274	296:300
L#48	OPR	409:445	275:319	246:254	299:327	252:264	285:301	326:346	267:295	252:276	239:251	278:294	379:399	266:266	224:228	248:252	284:300	266:278	296:328
L#49	OPR	425:429	279:303	238:238	315:327	264:296	281:293	346:346	267:299	256:260	263:263	282:286	379:383	262:266	228:228	228:260	284:308	266:266	312:320
L#50	OPR	433:445	303:303	254:258	307:319	264:264	273:273	326:342	283:295	244:252	239:251	282:294	391:399	254:266	224:228	248:260	312:312	266:266	300:300
L#51	OPR	433:445	283:291	238:254	299:319	264:276	261:293	330:346	243:267	248:248	243:251	290:294	383:387	254:254	228:232	228:244	280:288	266:294	300:328
L#52	OPR	429:445	283:283	246:246	327:327	276:296	277:293	326:334	279:291	252:256	251:251	286:290	399:403	270:278	228:228	236:260	288:312	266:270	312:320
L#53	OPR	409:429	279:283	246:246	315:327	264:264	273:285	326:342	267:283	256:260	243:263	294:294	391:395	254:286	228:240	268:268	288:304	270:294	300:328
L#54	OPR	425:433	275:299	258:270	299:323	264:276	269:277	326:342	267:283	252:260	243:251	282:286	395:395	266:278	228:248	260:260	288:304	266:266	312:328
L#55	OPR	433:445	303:303	254:258	299:307	264:264	257:285	334:346	279:283	244:252	239:251	278:294	391:399	266:286	224:228	252:260	284:304	266:266	296:300
L#56	OPR	425:433	299:323	238:246	299:319	276:296	281:301	326:334	243:263	252:260	251:251	286:286	391:403	274:278	228:228	248:260	288:312	266:266	328:332
L#57	OPR	429:433	275:319	238:246	311:323	264:264	261:269	334:338	243:267	252:252	259:263	286:290	391:399	266:278	228:240	236:248	288:288	266:294	324:328
L#58	OPR	425:433	283:319	238:258	319:323	264:276	277:281	334:334	259:287	256:260	239:251	286:290	383:399	262:262	224:228	248:260	284:300	274:278	292:328
L#59	OPR	429:433	275:279	246:246	323:327	276:276	257:269	334:338	283:295	256:260	243:255	286:286	391:403	246:286	228:240	252:260	288:288	270:274	320:332
L#60	OPR	429:433	279:299	238:246	311:315	264:296	261:269	338:338	283:299	248:248	251:263	286:286	391:395	266:270	228:228	260:268	280:288	266:294	328:328
L#61	OPR	433:445	283:295	246:254	319:327	276:296	261:281	326:326	271:287	248:256	243:255	286:294	379:383	266:266	224:232	244:260	288:288	266:286	300:328
L#62	OPR	409:429	303:319	246:254	299:327	252:264	285:301	326:346	279:287	252:260	239:251	278:282	395:399	254:266	224:228	244:252	288:300	266:278	300:320
L#63	OPR	425:429	283:283	246:246	307:311	276:276	273:293	326:334	259:279	248:256	251:251	290:294	379:403	254:278	228:228	236:260	288:312	270:294	320:332
L#64	OPR	425:433	299:319	246:246	315:327	264:264	257:277	334:346	259:267	252:252	239:243	286:294	391:391	278:286	224:240	236:252	296:304	270:274	300:332
L#65	OPR	425:433	283:299	238:246	315:327	264:264	261:277	334:338	275:275	264:264	243:251	286:286	395:399	266:266	228:236	248:268	288:288	274:278	292:300
L#66	OPR	433:445	319:319	246:246	323:327	264:296	257:317	326:346	271:287	248:252	251:251	282:290	391:399	266:266	228:228	232:260	288:288	000:000	300:312
L#67	OPR	425:429	279:319	246:270	323:323	296:296	269:277	334:338	243:259	248:260	243:243	286:286	383:391	266:278	240:248	236:260	288:292	266:266	312:312
L#68	OPR	429:429	279:319	246:246	323:327	264:296	257:293	322:334	243:259	248:252	243:255	286:290	391:391	278:286	232:240	260:260	000:000	000:000	312:324
L#70	OPR	433:445	295:319	246:246	307:323	264:296	257:257	322:326	243:259	248:252	251:251	290:290	391:399	266:266	228:228	248:260	284:288	274:278	296:324
L#71	OPR	429:433	295:319	246:246	323:327	264:296	261:281	322:326	271:287	252:260	243:251	282:290	391:395	266:266	228:228	232:260	000:000	266:278	300:324
L#72	OPR	433:433	275:279	246:250	299:307	264:264	261:269	322:326	259:267	248:252	239:243	286:290	387:391	266:286	224:236	228:256	288:292	266:274	312:328
L#73	OPR	429:445	283:319	246:246	307:327	264:296	257:293	322:326	279:283	252:256	239:243	286:290	391:403	270:278	224:240	260:260	000:000	000:000	320:328
L#74	OPR	429:433	283:319	238:246	307:323	264:264	257:317	322:326	243:287	248:256	251:259	282:290	391:399	266:266	228:240	232:260	288:288	270:294	300:312
L#75	OPR	429:429	279:319	246:246	323:327	272:296	257:269	322:334	259:283	248:252	243:243	286:290	391:391	278:286	240:240	260:268	288:292	266:294	312:328
L#76	OPR	429:433	319:319	246:246	323:327	296:296	261:281	322:326	259:271	248:252	243:251	290:290	371:399	266:266	228:240	248:260	288:288	274:294	296:312
L#77	OPR	429:433	283:283	246:254	323:327	276:296	273:293	322:326	243:291	252:252	243:243	290:294	379:399	254:266	224:240	236:244	288:292	286:294	300:312
L#78	OPR	425:429	295:319	246:246	323:323	264:296	261:293	322:326	243:283	252:252	251:255	282:290	391:391	270:278	228:232	232:260	280:288	000:000	000:000
L#79	OPR	425:429	279:295	246:246	323:327	272:296	261:269	322:334	259:283	248:252	243:251	282:290	391:391	278:278	228:240	232:268	280:292	278:294	312:324

Supplementary Table S5. Continued.

Individual ID	Location	LAT1	LAT2	LAT3	LAT4	LAT5	LAT6	LAT7	LAT8	LAT9	LAT10	LAT11	LAT12	LAT13	LAT14	LAT17	LAT18	LAT19	LAT20
L#80	OPR	429:445	283:283	254:258	319:327	264:276	261:293	326:334	243:259	248:248	251:251	286:286	379:383	254:254	228:228	228:260	288:288	286:294	300:320
L#81	OPR	433:433	275:295	246:246	319:323	264:276	269:269	334:338	287:295	260:260	239:255	286:286	375:391	246:270	224:228	252:268	288:296	270:274	300:332
L#85	OPR	429:433	283:283	246:254	311:327	276:296	273:293	326:338	259:291	248:252	243:251	290:294	379:399	254:278	224:228	236:244	288:316	266:294	312:332
L#86	OPR	425:433	275:299	246:246	299:315	264:296	261:281	326:338	243:275	248:264	243:255	286:290	383:399	266:266	232:236	260:268	284:288	270:274	292:332
L#87	OPR	429:429	319:319	246:246	311:323	264:296	273:293	326:338	259:271	248:252	243:251	286:290	391:391	266:278	228:240	236:268	288:292	266:294	312:324
L#88	OPR	429:429	295:319	238:246	299:311	264:296	269:293	326:338	283:295	252:252	243:259	286:290	391:391	266:278	240:240	236:260	288:292	266:266	324:328
L#89	OPR	433:445	275:283	246:254	307:327	264:296	257:293	326:338	243:267	248:252	243:263	290:290	379:399	254:254	224:228	236:268	276:288	266:286	312:328
L#90	OPR	425:433	299:299	238:246	299:315	264:264	277:281	326:346	267:295	252:256	239:251	286:286	391:391	262:278	224:228	252:260	288:296	270:270	300:320
L#91	OPR	425:433	275:275	254:258	299:327	276:276	269:269	334:334	267:299	244:268	251:251	286:290	379:391	246:274	228:228	248:252	288:312	270:274	324:328
L#92	OPR	425:433	283:283	246:246	307:311	276:296	277:293	326:326	259:279	252:256	239:251	290:290	391:399	254:278	224:228	244:248	288:288	270:294	296:332
L#93	OPR	425:433	299:323	238:246	299:327	264:264	269:277	326:346	267:295	252:256	243:251	286:294	391:391	262:278	228:240	236:260	296:308	270:270	328:332
L#94	OPR	425:429	303:319	246:258	323:327	276:296	269:277	334:346	259:263	248:260	251:255	286:290	383:391	262:266	228:232	248:268	288:300	274:294	296:328
L#95	OPR	425:429	295:295	246:246	315:323	264:296	257:261	322:338	259:275	252:260	251:251	286:290	391:395	246:278	228:228	232:268	288:292	274:278	328:328
L#96	OPR	425:433	275:279	238:246	299:327	264:276	257:301	326:334	267:283	248:252	239:239	286:286	391:403	270:286	224:224	260:268	288:312	266:266	320:332
L#97	OPR	433:433	295:319	238:246	323:323	264:296	261:317	326:338	243:271	252:256	251:259	290:290	371:391	266:266	228:240	232:248	288:288	278:294	312:324
L#98	OPR	425:433	283:299	246:246	299:315	264:264	277:301	338:338	275:275	256:264	239:243	286:290	391:399	266:266	224:236	248:268	288:296	266:274	300:328
L#99	OPR	433:445	275:319	246:262	307:327	264:296	269:317	326:346	243:271	252:268	251:251	286:290	371:403	266:266	228:228	232:260	288:288	270:294	296:324
L#100	OPR	429:433	279:279	246:246	299:327	276:296	269:281	334:334	259:263	248:252	243:251	286:290	391:391	274:278	228:240	260:260	292:312	266:294	328:332
L#101	OPR	425:429	283:319	246:246	323:327	264:296	257:277	334:334	283:283	248:256	243:251	286:286	391:403	270:286	228:240	248:260	288:312	270:294	320:328
L#102	OPR	429:433	283:319	238:246	307:323	264:264	281:317	322:338	259:271	248:256	251:259	290:290	371:399	266:266	228:240	232:260	284:288	266:294	300:312
L#103	OPR	429:429	275:295	238:246	315:323	276:296	273:301	338:346	259:287	248:252	243:259	286:290	391:391	246:266	240:240	248:260	288:288	270:270	300:312
L#104	OPR	429:445	283:319	246:246	327:327	272:276	269:277	334:334	279:283	252:256	243:251	290:290	391:403	254:278	228:240	248:268	288:292	266:270	320:328
L#105	OPR	433:433	275:295	238:246	299:299	264:296	257:301	326:326	267:295	248:252	239:251	286:290	387:403	270:270	224:228	228:268	288:312	266:266	320:332
L#106	OPR	409:445	303:319	246:254	299:319	264:264	273:285	326:346	279:295	244:276	239:251	278:282	379:399	266:266	224:228	248:260	284:312	266:266	296:300
L#107	OPR	433:445	283:319	246:246	323:327	264:264	281:317	322:338	271:287	256:260	243:251	282:290	371:395	266:266	228:240	232:260	284:288	266:294	300:324
L#108	OPR	409:429	291:299	238:258	319:327	264:276	257:261	334:346	283:299	252:260	251:259	282:290	379:383	266:270	228:228	244:268	312:312	266:266	292:328
L#109	OPR	425:425	283:299	238:246	315:323	264:296	257:277	326:338	275:283	252:264	243:251	286:286	395:399	266:286	228:240	236:248	288:304	266:274	292:328
L#110	OPR	425:433	275:299	238:246	299:315	264:276	261:269	334:338	267:275	260:264	243:243	286:290	391:399	266:278	228:236	248:260	288:288	270:278	292:328
L#111	OPR	425:433	283:323	246:246	315:319	264:276	261:281	326:334	243:275	260:264	000:000	000:000	000:000	266:278	232:236	248:260	288:288	266:278	292:332
L#112	OPR	425:429	295:319	246:246	323:327	272:296	257:261	322:334	283:283	252:252	243:251	290:290	391:391	270:278	228:240	260:260	280:292	266:266	312:328
L#113	OPR	433:445	275:283	246:262	311:327	264:296	269:273	334:338	243:267	248:252	243:251	286:286	383:399	254:278	228:240	232:260	292:300	266:294	312:328
L#114	OPR	425:433	299:323	238:246	299:319	264:296	269:281	326:334	283:295	252:256	251:255	286:286	375:391	262:266	228:232	260:260	288:308	270:274	320:328
L#115	OPR	421:429	295:323	246:246	315:323	264:296	261:293	322:346	243:263	248:252	251:255	286:290	391:395	266:270	228:228	232:236	280:288	266:270	312:320
L#116	OPR	433:445	275:295	238:246	299:307	264:296	257:281	334:338	267:283	248:252	255:263	286:290	391:399	254:266	228:232	268:268	276:288	266:266	328:332
L#117	OPR	429:433	279:295	238:246	299:327	264:276	293:301	326:334	283:295	248:252	239:243	286:290	391:403	270:286	224:240	228:260	288:288	266:270	320:332
L#118	OPR	429:429	275:303	246:246	315:327	296:296	269:269	326:346	263:283	248:252	255:263	286:290	383:391	266:270	228:232	260:268	288:312	266:294	296:332

Supplementary Table S5. Continued.

Individual ID	Location	LAT1	LAT2	LAT3	LAT4	LAT5	LAT6	LAT7	LAT8	LAT9	LAT10	LAT11	LAT12	LAT13	LAT14	LAT17	LAT18	LAT19	LAT20
L#119	OPR	425:433	279:295	246:254	307:319	276:288	273:273	326:338	279:283	256:272	239:243	286:290	375:395	266:290	224:232	244:260	280:288	270:278	296:328
L#120	OPR	429:429	275:319	246:246	323:327	296:296	273:281	322:334	243:267	248:252	243:263	286:290	383:399	254:266	228:240	236:260	292:300	294:294	296:312
L#121	OPR	429:433	283:319	238:246	323:327	296:296	261:281	322:326	243:259	256:260	251:259	290:290	371:395	266:266	228:240	248:260	288:288	266:294	296:324
L#122	OPR	429:429	295:319	238:246	299:311	264:296	269:293	326:338	283:295	252:252	243:259	286:290	391:391	266:278	228:240	236:260	288:292	266:266	324:328
L#123	OPR	433:433	000:000	238:246	299:327	264:296	257:269	326:334	267:283	252:252	239:251	286:286	391:391	266:286	224:228	236:260	288:304	270:274	320:332
L#125	OPR	429:433	295:319	238:246	307:323	264:296	261:281	326:346	243:259	248:256	243:259	290:290	371:399	266:266	240:240	232:260	288:288	274:294	296:324
L#127	OPR	425:433	299:323	238:246	315:319	264:296	257:269	334:334	259:295	252:252	239:251	286:294	391:391	262:278	224:228	252:260	296:308	274:274	320:332
L#128	OPR	429:433	275:279	246:246	299:327	276:296	257:301	326:334	267:283	248:252	243:251	286:286	391:403	270:286	228:240	228:260	288:288	266:270	320:332
L#129	OPR	429:429	283:291	254:258	299:319	264:276	261:269	326:334	243:259	248:248	243:251	286:294	379:395	254:254	224:228	244:260	288:288	286:294	300:328
L#130	OPR	425:429	279:283	246:246	323:327	276:296	257:293	334:334	259:279	248:256	243:251	286:286	391:403	270:286	228:240	260:260	288:288	266:270	320:328
L#131	OPR	409:425	000:000	246:254	323:327	264:264	293:301	326:334	283:295	252:260	239:251	290:290	391:395	254:270	224:228	244:260	280:288	274:278	300:324
L#132	OPR	425:433	279:295	238:246	299:327	264:272	257:257	326:334	283:295	248:256	239:243	286:290	387:403	254:270	224:240	260:268	288:288	266:266	320:332
L#133	OPR	429:429	295:319	246:246	323:327	272:296	257:261	326:334	283:283	248:252	243:255	290:290	391:391	270:278	232:240	232:268	288:292	266:278	312:312
L#134	OPR	429:433	295:319	246:246	311:323	264:264	261:269	322:338	243:271	252:256	243:243	282:286	395:399	266:278	240:240	232:236	288:288	266:294	300:328
L#135	OPR	425:433	283:295	238:246	315:323	264:296	257:261	326:334	275:283	260:264	243:251	286:286	391:395	266:286	228:240	236:268	288:304	278:294	292:328
L#136	OPR	425:425	275:299	246:246	299:327	264:276	277:281	334:334	243:275	248:264	243:255	286:290	383:395	266:278	232:236	260:268	284:288	266:278	300:332
L#137	OPR	425:429	275:295	246:270	323:323	296:296	269:269	338:346	243:259	248:260	243:243	286:286	383:391	254:262	240:248	236:260	284:296	266:274	312:312
L#138	OPR	429:433	283:319	238:246	323:327	296:296	257:261	338:346	271:287	256:260	243:259	290:290	371:399	266:266	240:240	248:260	288:288	274:294	296:324
L#139	OPR	429:445	000:000	246:246	307:323	264:296	000:000	322:326	279:283	248:256	000:000	000:000	000:000	270:278	228:240	248:268	292:312	266:270	296:328
L#140	OPR	433:445	295:295	238:246	307:323	296:296	261:281	322:326	259:271	252:260	251:259	290:290	371:395	266:266	228:240	232:260	288:288	266:294	300:312
L#141	OPR	433:445	275:291	254:258	299:319	264:276	277:293	330:346	243:259	248:248	251:251	286:286	000:000	254:254	228:228	260:260	288:316	266:294	300:328
L#142	OPR	425:433	295:299	246:246	315:327	264:264	257:261	334:338	275:283	260:264	000:000	286:286	391:395	246:266	228:236	268:268	288:288	274:294	300:328
L#143	OPR	425:433	275:299	254:258	299:327	276:276	269:269	334:334	267:299	244:268	251:251	286:290	379:391	246:274	228:228	248:252	288:312	270:274	324:328
L#144	OPR	429:429	295:319	246:246	323:327	296:296	257:261	322:334	243:283	248:252	243:255	286:286	391:391	278:286	232:240	232:260	288:288	266:278	312:328
L#145	OPR	429:445	279:283	246:246	327:327	264:272	257:293	334:334	283:283	248:256	239:243	290:290	391:403	270:278	224:240	248:268	292:312	266:270	296:312
L#146	OPR	409:429	275:283	246:254	311:327	264:296	285:317	338:346	243:267	252:252	239:251	286:294	391:399	266:266	224:228	248:260	288:300	278:278	324:328
L#147	OPR	425:433	275:319	238:246	299:311	296:296	257:273	326:326	259:295	252:252	251:259	286:286	391:403	266:270	228:240	260:268	288:312	266:270	324:332
L#148	OPR	425:429	279:295	246:246	323:323	272:296	269:293	322:326	259:283	252:252	243:251	290:290	391:391	270:278	228:240	256:260	280:292	266:266	312:324
L#149	OPR	433:433	307:307	238:246	299:307	264:296	269:273	334:338	295:299	248:264	243:255	286:290	391:399	254:274	232:240	260:260	288:288	274:294	292:328
L#150	OPR	429:429	319:319	246:246	311:323	264:296	273:293	326:338	259:271	248:252	243:251	286:290	000:000	266:278	228:240	236:268	288:292	266:294	312:324
